# The Organochlorine Pesticides Residue Levels in Karun River Water

**Published:** 2013-02-13

**Authors:** Abdolazim Behfar, Zahra Nazari, Mohammad Hassan Rabiee, Gholamreza Raeesi, Mohammad Reza Oveisi, Nafiseh Sadeghi, Behrooz Jannat

**Affiliations:** 1 Department of Drug and Food Control, Faculty of Pharmacy, Jundishapur University of Medical Sciences, Ahvaz, IR Iran; 2 Department of Pharmacology and Toxicology, Pharmacy School, Jundishapur University of Medical Sciences, Ahvaz, IR Iran; 3 Central Laboratory of Isfahan Water and Wastewater Company, Isfahan, IR Iran; 4 Company of Ahvaz Water and Sewage, Water and Wastewater Management, Quality Control and Health Monitoring , Ahvaz, IR Iran; 5 Department of Drug and Food Control, Tehran University of Medical Sciences, Tehran, IR Iran; 6 Water Research Center, Ministry of Health, Tehran, IR Iran; 7 Food and Drug Laboratory Research Center, Ministry of health, Tehran, IR Iran

**Keywords:** Organochlorine Pesticide, Water, Karun River, Iran

## Abstract

**Background:**

The organochlorine pesticides (OCPs) are among the most commonly used in water streams around the world. Most of these contaminants are highly hydrophobic and persist in sediments of rivers and lakes. Studies have suggested that OCPs may affect the normal function of the human and wildlife endocrine systems.

**Objectives:**

The aim of this study is to determine the concentrations of selected organochlorine pesticides residues [OP'DDT, PP'DDT, alderin, dieldrin, heptachlor, (α,ß,γ,δ) HCH, (α, ß) endosulfan and metoxychlor] in samples from Karun River water at Khuzestan province in Iran , by GC-µ-ECD.

**Materials and Methods:**

Water was extracted with n-hexane and then purified by passing through a glass column packed with Florisil and Na_2_SO_4_, which was then eluted with ether: hexane solution v/v.

**Results:**

In general, all of 12 investigated organochlorine pesticides (OCPs) were detected. Regardless of the kind of OCPs, the highest OCP pollution level in Karun River were seen from August to November 2009 ranging 71.43 – 89.34 µg/L, and the lowest were seen from Dec 2010 to March 2011 at levels of 22.25 - 22.64 µg/L. The highest and lowest mean concentrations of 12 investigated pesticides were ß-Endosulfan and pp' DDT with 28.51and 0.01 µg/L respectively.

**Conclusions:**

Comparison of total organochlorine pesticides residues concentration with WHO guidelines revealed that the Karun River had total OCPs residues above the probable effect level (0.2-20 µg/L, P < 0.05), which could pose a risk to aquatic life.

## 1. Background

The organochlorine pesticides (OCPs) such as dichlorodiphenyltrichloroethanes (DDTs), hexachlorocyclohexanes (HCHs) and endosulfans are among the most commonly used pesticides in water streams around the world (1, 2). Most of these contaminants are highly hydrophobic and persist in sediments of rivers and lakes which facilitate their accumulation in lipid-rich tissues of biota (3-6). Their widespread use combined with over-application, accidental spills, runoff from mixing-loading areas, and faulty waste disposal creates environmental concerns as well as toxic biological effects (7, 8). Studies have suggested that OCPs may affect the normal function of the human and wildlife endocrine systems (9).

International consensus is growing for restored management of water and its quality. Monitoring of threatening organic pollutants in surface water is essential to attain high quality water. The amounts of pesticides in surface waters depends on several factors including soil characteristics, topography, agricultural management practices and chemical and environmental properties of individual pesticides. The safety and physicochemical properties of some OCPs are shown in ( Table 1 ).

**Table 1:** The Physicochemical and Safety Properties of Some Selected OCPs

Pesticides	K, mg.g^-1^	Half Life, d	GV, µg.L^-1^	TDI %	HV, µg.L^-1^	MCL, µg.L^-1^	MAVs, µg.L^-1^
**Lindane **	1100 1161	400	2 (WHO) 0.05 (AUS)	1 (WHO)	20 (AUS)	0.2 (US)	2 (NZ)
**Heptachlor **	24000					0.4 (US)	
**Heptachlor epoxide **						0.2 (US)	
**Heptachlor + Heptachlor epoxide**			0.03 (WHO) 0.05 (AUS)	1 (WHO)	0.3 (AUS)		0.04 (NZ)
**Aldrin/Dieldrin**	8400		0.03 (WHO) 0.01 (AUS)	1 (WHO)	0.3 (AUS)		0.03 (NZ)
**Endosulfan **	12400	50	0.05 (AUS)		30 (AUS)		
**DDT**	243000		2 (WHO) 0.06 (AUS)	1 (WHO)	20 (AUS)		2 (NZ)
**Chlordane**	38000		0.2 (WHO) 0.01 (AUS)	1 (WHO)	1 (AUS)	2 (US)	0.2 (NZ)
**Methoxychlor**			20 (WHO) 0.2 (AUS)	10 (WHO)	300 (AUS)	40 (US)	20 (NZ)

Abbreviations: GV, Guideline values; TDI, Tolerable daily intake; HV, Health value; MCL, Maximum contaminant Level; MAV, Maximum acceptable values

## 2. Objectives

The aim of this study was to determine the concentrations of selected organochlorine pesticides residues: OP'DDT, PP'DDT, alderin, dieldrin, heptachlor, (α,ß,γ,δ) HCH, (α, ß) endosulfan and metoxychlor in Karun River water samples at Khuzestan province in Iran , by GC-µ-ECD as well as to characterize the relationships among the OCPs species, and to carry out a partial validation of methodology.

## 3. Materials and Methods

### 3.1. Chemicals

All solvents (n-hexane, petroleum ether, diethyl ether, and acetone) were pesticide residue analysis grade with 98-99% purity purchased from Merck Co., Germany. The solvent was consumed to evaluate 12 components: OP'DDT, PP'DDT, alderin, dieldrin, heptachlor, (α,ß,γ,δ) Hexachlorocyclohexan (HCH), (α, ß) endosulfan and metoxychlor. Organochlorine pesticides standards were obtained from Dr. Ehrenstorfer GmbH (Germany). Working solutions were prepared in hexane at 1 μg mL^−1^. Florisil 60–100 mesh as the sorbent material obtained from Sigma–Aldrich (UK), activated following drying at 150º ^C^ about 12 hours prior using.

All glassware was carefully washed with soapy water, rinsed with distilled water ethanol, acetone and pesticide grade n- hexane respectively.

### 3.2. Sampling

**Figure 1:**
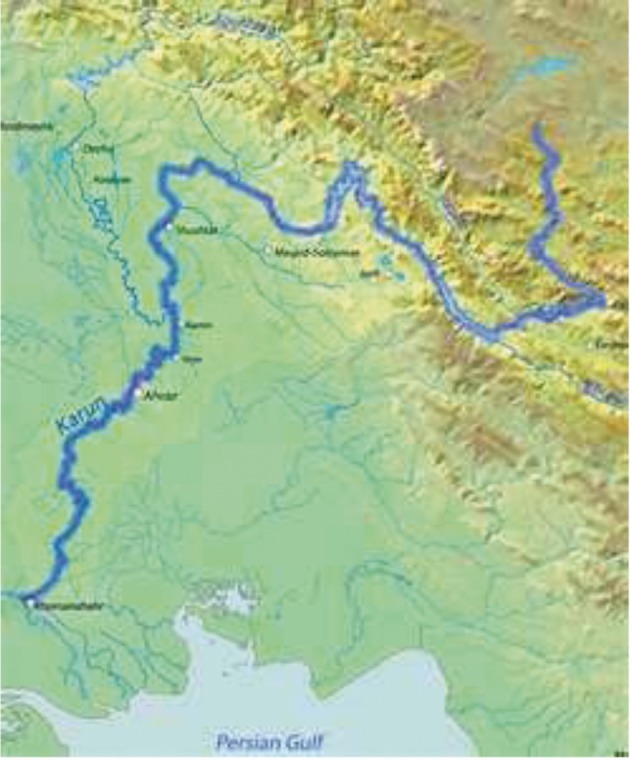
Location of the Study Area Showing the Karun River

The selected region for the pesticides monitoring survey was Karun River in the province of Khuzestan ( Figure 1 ). Khuzestan province has some major cities and is in an area with sugar/alcohol power plants and oil that suffers an impact both industrially and agriculturally. The Karun River extends 900 Km as one of the largest rivers flowing into the Persian Gulf. Its average annual flow is 702 ,m3s^-1^ in which its agricultural, industrial and urban sewerage load is 41.6, 10.871 and 5.25 m3s^-1^, respectively. The major agricultural basin comprises 1250 Km² and has wheat, corn, and sugarcane farms.Sampling was performed monthly from August 2009 to March 2011 in order to investigate the contaminants qualification and quantification in Karun River. The water samples are from eight locations along the river (Dez entrance, Shatit entrance, Gargar entrance, entry to Mollasany, entry to Ahvaz city, Ahvaz refinery entry, emersion of sediment pool and emersion of Ahvaz refinery). Samples were collected on a monthly basis, four times a day, from 3 parts in width of the river at a depth between 5-15 cm using 1 L precleaned amber glass bottles. Following combining the 15-20 samples as well as preparing the mix sample “in situ”, water was acidified by sulfuric acid to PH 2 and filtered through glass fiber filters in order to eliminate biological activity and particles. The filled amber bottles were subsequently transported to the laboratory and kept at 4°^C^ in the dark until further extraction and analysis.

### 3.3. Gas Chromatography

The water (250 mL) was extracted three times with 20mL n-hexane .The extracts were combined and concentrated using a rotary vacuum evaporator to 10 mL. Extracts were purified by passing them through a glass column (20 cm, 0.8 mm id) packed with florisil (10 cm) and Na_2_SO_4_ (2 cm), which was then eluted with ether/hexane solution; 200 mL at 6:94 v/v, followed by 15:85 v/v and 50:50 v/v. Thereafter, elutes were evaporated to dryness using a rotary vacuum and the residues were dissolved in 10 mL hexane for gas chromatography analysis. Spiked matrix samples were analyzed as part of QA/QC. Analysis was ended using an Agilent 6890N (HP) gas chromatograph with a micro-cell electron capture detector (µ-ECD). A DB-5 column 30 mm ×0.25mm i.d., containing 5% phenyl-methylpolysiloxane with a phase thickness of 0.25 μm. The temperature program used for the analysis was: 210°^C^ to 275°^C^ (13min) at 4°^C^ min^−1^. The injector and detector were set to 300°^C^ and 320°^C^ in the split less mode. Nitrogen was used as the carrier at 0.7 mL min^−1^ and the make-up gas at 35 mL min^−1^. Identification of peaks was based on the comparison of the retention times of compounds in the standard solutions. Quantification of the analyzed compounds was performed using the method of the internal standard (10).

## 4. Results

Retention times, calibration equations and coefficients of calibration (R2) of determined pesticides had been performed followed by GC; electron capture detector. The correlation coefficients were indicating good performance of the chromatographic method.

Recoveries were obtained with distilled water samples spiked with standards ( Table 2 ), which ranged from 84% to 98%. Minimum detection limits were calculated by using a signal-to-noise ratio of 3 ( Table 2 ). These parameters are in beneficial range of the accuracy of 70–130 % and precision of 30 % for trace level organic compounds in water samples, according to the US–EPA methods general characteristics (11). Monthly concentrations of organochlorine pesticides detected in Karun River are shown in Table 3.

In general, higher total organochlorine pesticides residues were found in the upper Karun River as compared with those in the lower sites; and they decrease significantly along the river due to the sediment adsorption, dilution or natural refinery, ranging between 24.48 to 3.38 µgL^-1^.

**Table 2:** Recoveries and Minimum Detection Limits (MDL) of Organochlorine Pesticide (OCPs) Standards Spiked to Karun River Water

OCPs	Spiked at μg/ L	% Recovery	MDL, μg/ L	CV
**α-HCH**	0.025	94	0.0022	4.48
**β-HCH**	0.01	95	0.0036	7.27
**γ-HCH**	0.015	86	0.004	5.6
**δ-HCH**	0.01	90	0.002	9.38
**Heptachlor**	0.01	89	0.0015	8.5
**Aldrin**	0.01	88	0.007	5.17
**Dieldrin**	0.02	84	0.009	4
**α-Enosulfan **	0.015	86	0.0088	5.79
**β-Enodosulfan **	0.035	94	0.008	5.15
**OP' DDT**	0.015	98	0.001	6.9
**PP' DDT**	0.01	90	0.0019	10.1
**Metoxychlor**	0.05	98	0.022	7.5

**Table 3:** Monthly Concentrations of Organochlorine Pesticides (OCPs) Detected in Karun River Water Samples (µg/L)

OCPs	Month					
	Aug 2008	Sep 2008	Oct 2008	Nov 2008	Dec 2008	March 2009
**α - HCH**	0.0764	0.1165	0.0113	0.0104	0.0082	0.2356
**β - HCH**	6.0681	3.4905	0.0822	0.2265	0.2103	0.8087
**δ - HCH**	1.2888	3.2621	0.8347	2.4896	0.2875	0.5505
**γ - HCH**	2.8766	4.2528	0.6456	0.7932	0.2259	0.7167
**Total HCH ^a^**	10.3098	11.1219	1.5739	3.5196	0.7319	2.3115
**Heptachlor**	4.9873	2.7526	1.1380	9.0000	5.1627	2.0798
**Aldrin**	1.1542	1.1886	0.5647	0.2583	0.1390	1.4749
**Dieldrin**	1.6034	1.7756	0.9121	0.8192	0.1376	0.8120
**Total Aldrin ^b^**	2.7576	2.9642	1.4767	1.0775	0.2766	2.2869
**α - Endosulfan**	22.7222	20.1273	18.6447	24.2042	6.2346	2.2630
**β -Endosulfan**	33.6993	31.8974	44.5903	49.8250	9.4587	1.5900
**Total Endo ^c^**	56.4215	52.0248	63.2350	74.0292	15.6933	3.8530
**OP' DDT**	0.0364	0.0873	0.0323	0.0000	0.0000	0.0000
**PP' DDT**	0.0280	0.0030	0.0024	0.0074	0.0059	0.0146
**Total DDT ^d^**	0.0645	0.0903	0.0348	0.0074	0.0059	0.0146
**Metoxychlor**	10.6786	2.4783	5.3250	1.7073	0.7701	11.7049
**Total OCPs ^e^**	85.2192	71.4320	72.7833	89.3409	22.6406	22.2507

^a^Total HCH (α - HCH + β - HCH + δ - HCH + γ - HCH)

^b^Total Aldrin (Aldrin + Dieldrin)

^c^Total Endo (α + β Endosulfan)

^d^Total DDT (OP' + PP' DDT)

^e^Total OCPs (Total of 12 investigated Organochlorine Pesticide residue regardless the kind of them)

## 5. Discussion

Comparison of total organochlorine pesticides residues concentration with WHO guidelines revealed that the Karun River had total OCPs residues above the probable effect level (0.2-20 µg/l; P < 0.05), which could pose a risk to aquatic life. The highest and lowest concentration of 12 OCPs belonged to ß–Endosulfane and pp' DDT, 28.51 ± 7.82 and 0.03 ± 0.01 µg/L, respectively. At the first glance on the linear regression analysis, a good linear correlation is seen between total OCPs levels and β, γ, total HCH, heptachlor, dieldrin, total Aldrin, total α, β-endosulfan and Metoxychlor.

The OCPs levels in the entry to the Ahvaz does not merit a significant difference with the emersion of sediment pool water and the emersion of Ahvaz refinery site, though the toxins level are decreased. Regardless of the type, the highest OCPs pollution level in Karun River were seen from August to November 2009 ranging 71.43–89.34 µg/L and the lowest were seen from Dec 2010 to March 2011 at levels of 22.25-22.64 µg/L. It means that levels of OCPs are decreasing from summer to winter, and possibly it is due to more farming in the summer.

In this study, data analysis of each OCPs pesticide shows that:

**Table 4:** Mean, SEM, (Standard error of mean) Minimum and Maximum of Organochlorine Pesticides (OCPs) Detected in Karun River Water Samples ( µg/L)

OCPs	Mean	SEM	Minimum	Maximum
**α - HCH**	0.0764	0.0366	0.0082	0.2356
**β - HCH**	1.8144	1.0002	0.0822	6.0681
**δ - HCH**	1.4522	0.4806	0.2875	3.2621
**γ - HCH**	1.5851	0.6556	0.2259	4.2528
**Total HCH ^a^**	4.9281	1.8710	0.7319	11.1219
**Heptachlor**	4.1867	1.1626	1.1380	9.0000
**Aldrin**	0.7966	0.2249	0.1390	1.4749
**Dieldrin**	1.0100	0.2439	0.1376	1.7756
**Total Aldrin ^b^**	1.8066	0.4265	0.2766	2.9642
**α - Endosulfan**	15.6993	3.7419	2.2630	24.2042
**β -Endosulfan**	28.5101	7.8293	1.5900	49.8250
**Total Endosulfan ^c^**	44.2094	11.4065	3.8530	74.0292
**OP' DDT**	0.0260	0.0141	0.0000	0.0873
**PP' DDT**	0.0102	0.0040	0.0024	0.0280
**Total DDT ^d^**	0.0362	0.0141	0.0059	0.0903
**Metoxychlor**	5.4440	1.9254	0.7701	11.7049
**Total OCPs ^e^**	60.6111	12.3956	22.2507	89.3409

^a^Total HCH (α - HCH + β - HCH + δ - HCH + γ - HCH)

^b^Total Aldrin (Aldrin + Dieldrin)

^c^Total Endo (α + β Endosulfan)

^d^Total DDT (OP' + PP' DDT)

^e^Total OCPs (Total of 12 investigated Organochlorine Pesticide residue regardless the kind of them)

### 5.1. HCH

α, β, δ, γ-HCH concentration is the highest in the primary of the river , but decrease significantly in the emersion of the sediment pool at Ahvaz refinery because of surface adsorption and infiltration. Total HCH is higher than allowable in the Mollasany location, though it is not noteworthy.

There is a positive correlation between total HCH concentration and heptachlore in the emersion of the refinery site, but it is inversed and negative for total Aldrin. Also in the location of sediment emersion pool, total HCH is correlated to each of α, β and δ- HCH isomers.

### 5.2. Heptachlor

Heptachlor pollution is significantly (P < 0.05) higher than WHO guideline value (GV= 0.03µg/L) in all of the sampling location of the Karun River.

### 5.3. Aldrin

This toxin level is upsurged along the Karun River but in sediment and refinery emersion sites it is definitely reduced. Though its level is further than allowed (GV = 0.03µ/L), it will be at a safe level following filtering.

### 5.4. Dieldrin, α and β-Endosulfan

Dieldrin, α and β-Endosulfan are at excessively high level at the primary location of the river. They are above the guidelines in three locations; Mollasany, Ahvaz city and the sediment pool emersion. Although at the refinery site the levels decreased significantly, which are not over permissible limits.

Total Aldrin and Endosulfan levels have linear correlation with Aldrin, Dieldrin and α, β- Endosulfan concentration in total samples of the river, respectively.

### 5.5. DDT

OP-DDT has decreased along the river since all river samples are lower than permitted value. PP-DDT has increased in the entry of Ahvaz city, which may be due to new pollution from the lower margin of the river or it could have been produced from DDT metabolism. There is significant correlation between OP-DDT and total DDT in Total River, but changes of PP-DDT concentration do not have a relationship with any other toxins.

### 5.6. Methoxychlor

There is no detected value of methoxychlor over permissible limits in each of the site river samples.

Moghaddam zand et al. in a research on Karun River in 2001 showed that DDT, Dieldrin and Aldrin is at trace amount in Ahvaz tap water, but in Karun River surface is in range of 0.013- 9.2 µ/L (10).

Organic contaminants are ubiquitous in surface environments through the world. Several studies have been devoted to investigating the fate of pesticides residue in the soil, water and air system. Gunderson et al indicated that 5 of 10 Ohio River paddlefish egg samples exceed the Food and Drug Administration's action limit for chlordane (12). Ouyang et al investigated the characteristics and spatial distribution of total chlordane, and its three most abundant compounds, including α– chlordane, γ-chlordane and trans–nonachlor in sediments from the Cedar and Ortega rivers, Florida, USA. They showed that two areas, one from the Cedar River and the other from the northern end of Ortega River area, were contaminated; which when compared to the Assessment Guidelines, total chlordane concentration in each area was above the probable effect level (13). Also the Fishweir Creek area has been identified by the St. Johns River Water Management District as a contaminated area including pesticides (14).

Azevedo et al. studied the Paraiba do Sul River, in the state of Rio de Janeiro for its water quality, by determining the levels of selected polycyclic aromatic hydrocarbons and pesticides from six sites in two cities, Resende and campos dos Goytacazes with industrial and agricultural activities. The detected compounds were atrazine and Irgarol (triazine pesticides) in downtown of compos dos Goytacazes and benzo [α] pyrene in Resende. The detected concentration values were around 0.2 µg/L (10). In Europe the regulation for pesticide has been set at 0.1µgL^-1^ and for polyaromatic hydrocarbons regulated at 0.2 µg/ L (15).

Gascon et al researched some organonitrogen herbicides, atrazin, alachlor and metalachlore in the Ebro River, applying Enzyme–linked immunosorbent assay (ELISA) and Gas chromatography mass spectrometry. The report of the study reveals that Atrazin was found to be the pesticide exhibiting the highest concentration throughout the year, and annual loads of organonitrogen herbicides were directly correlated with field application rates and stream discharge and the physicochemical properties of the herbicides (16).

OCPs were determined in Deep Bay; an important water body between Hong Kong and mainland China. The average concentrations of DDTs, HCHs and chlordanes in water were 1.96, 0.71 and 0.81 ngL^-1^. Temporal trends of the targeted OCPs levels in sediment core generally increased from 1948 to 2004, with the highest levels in top or sub-surface of the river. The risk assessment indicated that there were potential ecological and human health risks for the target OCPs in Deep Bay (17).

This study demonstrates that: higher total organochlorin pesticides residues which were found in the upper Karun River as compared with those in the lower sites; and they decrease significantly along the river due to the sediment adsorption, dilution or natural refinery, ranging between 3.38 to 24.48 µg 1^-1^. Comparison of total organochlorine pesticides level with WHO guide value showed that the Karun River had total OCPs residues above the probable effect level (0.2-20 µg/l; P < 0.05), which could pose a risk to aquatic life, and makes it necessary to keep a periodic approach to monitor OCPs, in Karun River.
